# What is wrong with my vision, and what can I do?

**Published:** 2012

**Table:** 

No eye disease	Glaucoma or ‘black blindness’	Cataract or ‘white blindness’
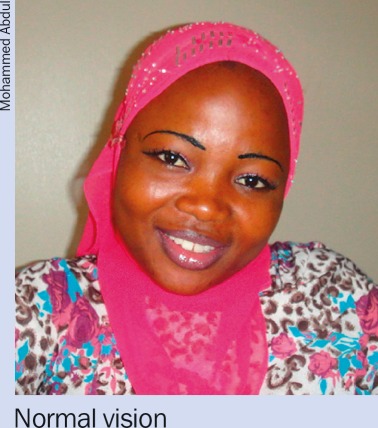	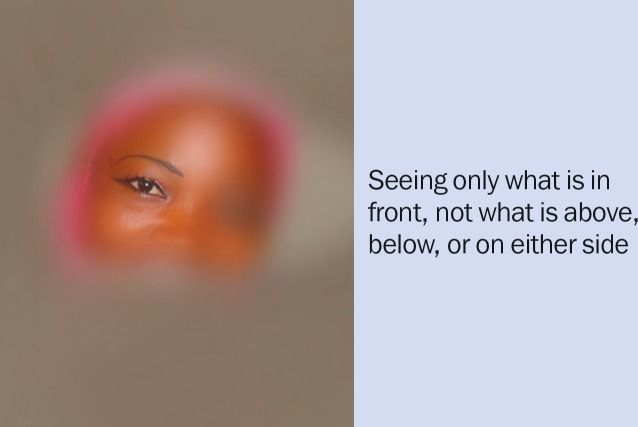	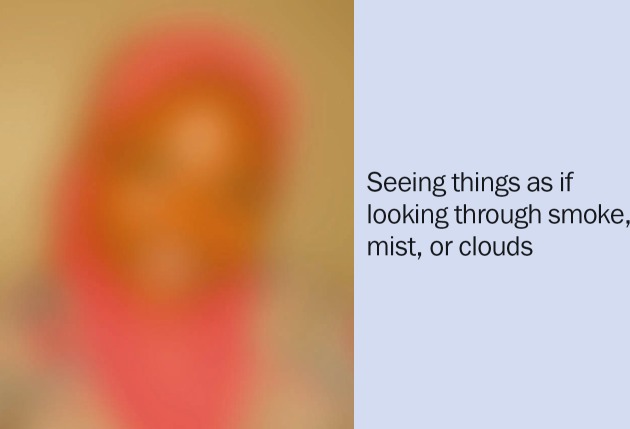
**Appearance of the eye to others**	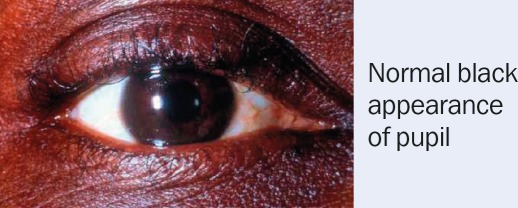	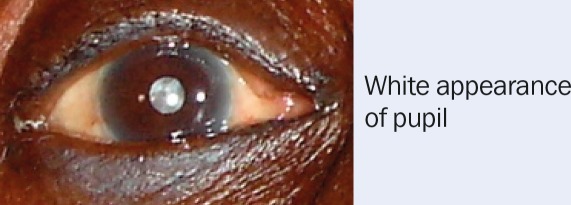
	**Glaucoma**	**Cataract**
**What can I notice/feel?**	Nothing initially, not even pain. You may often bump into things, or fall over objects on the ground, because you are losing the outer edges of your vision	Gradual clouding of the vision until the vision has almost gone. No pain. Usually in both eyes
**Who is at risk?**	People who are 40 years of age or older People with a relative who has glaucoma – a parent, sister or brother, or older child People who wear spectacles to see distant objects, who have had an eye injury before, or who use steroid eye drops.	People who are 40 years of age or older People with a previous eye injury People with diabetes People who use steroid eye drops or tablets
**Can other family members be affected?**	Yes! Glaucoma can run in the family	Cataract does not run in the family, but other older family members may also develop cataract
**How urgently do I need to get help?**	Very urgently!	As soon as possible
**What happens if I wait a long time before being treated?**	**You may lose your sight, and you will be unable to get it back**	It is very likely that you will regain vision
**What treatment options do I have?**	Vision can be **preserved** by lowering eye pressure with eye drops, an operation, laser treatment, ora combination of these options	Vision can be restored with a cataract operation and an artificial lens implant
**What long-term treatment may I need?**	Continued use of eye drops or monitoring after the operation or laser treatment at an eye clinic	Usually none. You may need to wear spectacles after the operation
**What are the costs?**	Medical treatment: the lifetime cost of eye drops An operation or laser treatment: the cost of an operation and possibly some medication afterwards	One-time cost of the cataract operation
**What are the risks of treatment?**	Very few side effects of eye drops. Surgery can have complications, but these can be managed at the eye clinic	Highly successful operation with very few or no complications
**What will happen if I stick to my treatment and/or say yes to an operation?**	Your vision will be preserved – you will still be able to see as you did before It will take a lot longer before you go blind, if you go blind at all	Your vision will be better than before
**What happens if I do not accept treatment?**	Your vision will gradually worsen and eventually you will become completely blind. **This vision is lost forever and can never come back!**	Your vision will gradually worsen until you become completely blind. But, at any time, accepting a cataract operation will restore your sight
**Will traditional medicine help me?**	No. Delay in obtaining the correct treatment means you are likely to lose even more vision	No. Traditional treatment, known as ‘couching’ (pushing a needle into the eye), can have very serious complications and is not recommended. Eye medication from traditional healers cannot restore sight
**What can I do?**	Report to the nearest eye clinic urgently to be examined. If you think a relative may have glaucoma advise them to do the same	Report to the nearest eye clinic to be examined. If you know someone who may have cataracts advise them to do the same

